# Chronic Pain in Spanish Physiotherapy Practice: Treatment Challenges and Opportunities in Diverse Healthcare Settings—A Qualitative Study

**DOI:** 10.3390/clinpract14050165

**Published:** 2024-10-10

**Authors:** Ángeles Díaz-Fernández, Esteban Obrero-Gaitán, Irene Cortés-Pérez, Ana Raquel Ortega-Martínez, María Catalina Osuna-Pérez, Noelia Zagalaz-Anula, María del Rocío Ibancos-Losada, Rafael Lomas-Vega

**Affiliations:** 1 Department of Health Sciences, University of Jaen, Campus las Lagunillas s/n, 23071 Jaen, Spain; andiaz@ujaen.es (Á.D.-F.); icortes@ujaen.es (I.C.-P.); mcosuna@ujaen.es (M.C.O.-P.); nzagalaz@ujaen.es (N.Z.-A.); mibancos@ujaen.es (M.d.R.I.-L.); rlomas@ujaen.es (R.L.-V.); 2 Department of Psychology, University of Jaen, Campus las Lagunillas s/n, 23071 Jaen, Spain; arortega@ujaen.es

**Keywords:** biopsychosocial approach, chronic pain management, physiotherapy, physiotherapists’ experiences, qualitative study

## Abstract

**Background/Objectives:** Physiotherapists face significant challenges in managing chronic pain; a complex condition best addressed through a biopsychosocial (BPS) approach. Although substantial evidence exists globally, research specific to Spain remains limited. This study explores the experiences and challenges Spanish physiotherapists encounter in integrating BPS elements across public and private healthcare settings. **Methods:** Semi-structured interviews with 12 experienced physiotherapists were conducted, using a qualitative approach with purposive sampling and reflexive thematic analysis. The analysis, grounded in a constructivist/interpretivist framework, allowed themes to emerge naturally from the data, supported by relevant quotes. **Results:** Three primary themes were identified: (1) challenges in implementing the BPS approach, including patient resistance and limited training; (2) facilitators for adopting the BPS model, such as multidisciplinary support and positive patient outcomes; and (3) emotional and psychological aspects of the physiotherapists. The findings highlight the complexities of chronic pain management in physiotherapy, emphasizing the need for enhanced psychological training, integrated multidisciplinary care, early diagnosis, and effective family involvement. These insights are critical for improving both patient care and physiotherapist well-being. **Conclusions:** This study provides valuable guidance for future strategies, focusing on educational enhancements, multidisciplinary collaboration, healthcare policy reform, and emotional support for physiotherapists within the Spanish healthcare context.

## 1. Introduction

Musculoskeletal disorders, particularly chronic pain, represent a significant global health challenge, often leading to substantial personal and societal costs [1,2,3]. Chronic pain, defined as pain persisting or recurring for over three months, is frequently accompanied by emotional distress and functional disability [4,5]. It is increasingly acknowledged that psychosocial factors including cognitive, emotional, behavioural, and social elements, play a crucial role in the persistence and intensity of chronic pain [6,7]. Consequently, clinical practice guidelines encourage a biopsychosocial (BPS) approach to managing these conditions, emphasizing the need for a comprehensive understanding of pain as an interaction of biological, psychological, and social factors that are unique to each patient [8,9].

Physiotherapists working in primary care, specialized hospital settings, or private clinics are often the first point of contact for patients with musculoskeletal complaints, placing them at the forefront of early intervention and management [10,11]. Their role extends beyond traditional biomechanical models, including identifying patients at risk of chronicity, screening for complexity in chronic pain cases, and providing tailored treatments based on the BPS model [12,13]. Despite the recognized importance of the BPS approach in physiotherapy, evidence suggests a gap between guideline recommendations and actual clinical practice [14,15]. Many patients continue to receive care that does not fully integrate the BPS model, highlighting a need for deeper insight into the barriers and challenges physiotherapists encounter in adopting this approach [16,17].

There is extensive international scientific literature on the subject, which identifies numerous difficulties in implementing the BPS approach and several facilitators to improve the treatment of patients with chronic pain [16,17,18,19,20,21,22]. However, there is a lack of research specifically addressing these issues in Spain, where studies on the barriers and facilitators faced by Spanish physiotherapists in adopting the BPS model are comparatively limited [23,24,25]. A recent study has indicated that, while physiotherapists in Spain possess adequate theoretical knowledge about chronic pain and its BPS management, many report a lack of confidence in their clinical practice [26]. This gap between knowledge and practice underscores the need for a more in-depth exploration of these challenges, highlighting the value of a qualitative perspective to understand the barriers and facilitators in adopting this BPS approach. In contemporary health research, qualitative methods are increasingly recognized for their efficacy in comprehensively exploring patients’ and healthcare professionals’ experiences, perspectives, and interpretations regarding illness or well-being [27]. These methods generate novel insights and advance understanding, making them highly valuable to researchers and health practitioners [16,17,18,20].

Therefore, through a qualitative approach, this study investigates potential barriers, facilitators, challenges, or differences physiotherapists encounter when treating chronic pain patients within a BPS orientation in the Spanish healthcare system (including primary care, specialized hospital care, and private clinics). By exploring the unique professional experiences and challenges physiotherapists face, this study aims to contribute to a deeper understanding of the barriers and facilitators in adopting the BPS model in diverse healthcare environments, with the goal of informing future strategies for better patient care and professional satisfaction among physiotherapists.

## 2. Materials and Methods

The research team employed semi-structured individual interviews with a diverse group of physiotherapists specialized in chronic pain management. The methodology and reporting of this study followed the Standards for Reporting Qualitative Research [28].

### 2.1. Setting and Participant Selection

The public healthcare framework in Spain is organized into two main sectors: primary care, encompassing services including general practitioners and physiotherapists; and specialized care, which includes hospitals, emergency services, and specialized centers, with varying access to resources and multidisciplinary support. General practitioners often serve as the first point of healthcare contact, offering free access and guiding patients through the healthcare journey, including referrals to specialized care. For physiotherapy services within primary care, referrals from general practitioners are common, though the public sector may sometimes have waiting lists. In recent years, there has been a shift towards enabling direct access to physiotherapy services, mainly through private healthcare channels. Spanish regulations for direct access to physiotherapy do not mandate additional specialized training or certification for physiotherapists.

In this qualitative study, physiotherapists specializing in chronic pain management across Andalucía (Southern Spain) were contacted through professional networks and peer recommendations. Invitations to participate in the study were sent via email. The participant pool was deliberately diverse in characteristics such as gender, age, and professional experience—with a minimum of 10 years of clinical experience in chronic pain management—to ensure a representative sample. Out of 18 physiotherapists initially approached by email, 14 consented to participate; the remaining six were unable to join due to scheduling conflicts, with no outright refusals. The selection of four physiotherapists from each sector (primary care centers, specialized hospitals, and private clinics) occurred randomly based on the availability of participants and was not pre-planned. These physiotherapists were considered experts in chronic pain management due to their extensive clinical experience, peer recommendations, and professional profiles shared via social networks. Purposeful sampling was employed to select participants with specialized knowledge in chronic pain, which aligns with the exploratory nature of qualitative research aimed at obtaining in-depth insights into specific phenomena.

### 2.2. Ethical Considerations

Before participation, all physiotherapists received detailed information about the study and signed an informed consent form in accordance with the ethical guidelines outlined in the Declaration of Helsinki [29]. The consent form outlined the study’s objectives, procedures, potential risks, and benefits; ensuring participants were fully informed. Participants were assigned unique codes to maintain confidentiality, and any identifying information was removed from the transcripts. Data were securely stored, and access was restricted to the research team. The study protocol was approved by the Ethics Committee of the University of Jaén (Reference No. 20240115/ENE.PRY).

### 2.3. Approach and Paradigm

In this research, the principal investigator, A.D.F., a physiotherapist PhD candidate at the time of the study, contributed to several studies on chronic pain in physiotherapy practice [26,30,31]. The interviews in this study are part of a larger mixed-methods research project focused on exploring the practical challenges physiotherapists encounter when managing patients with chronic pain and analyzing their knowledge, attitudes, and the various difficulties they face in clinical practice [26]. Based on the findings of a preceding study, physiotherapists in Spain possess adequate knowledge about chronic pain, but do not consistently implement the BPS approach in their practice. Furthermore, they did not report high levels of self-confidence or perceived skills regarding the treatment they provide to patients with chronic pain [26]. These insights have significantly shaped the approach and perspective of A.D.F. in conducting the current qualitative study. The awareness of these challenges among physiotherapists has guided the formulation of interview questions and has influenced the overall direction of the investigation.

The methodological approach of this study is rooted in constructivism/interpretivism, aligning with the principles of narrative research [32,33]. This approach emphasizes understanding physiotherapists’ unique perspectives and experiences in managing chronic pain. While our study is primarily constructivist, recognizing the subjective interpretations of each participant, we also integrate elements of critical realism. This dual approach recognizes that, while physiotherapists’ experiences have an objective reality, they interpret it through their perspectives. By navigating the intersection of these paradigms, we value both the objective aspects of physiotherapists’ experiences and their subjective interpretations [32,33]. Our analysis of the semi-structured interview content employed a reflexive thematic approach, focusing on identifying, analyzing, and reporting patterns or themes that emerged from the data, until saturation was reached [34]. This process involved a thorough and repeated review of interview transcripts to generate initial codes. These codes were then grouped into themes and carefully reviewed to ensure they coherently and accurately represented the data collected [34]. We used an inductive methodology to categorize and code data, allowing categories and themes to emerge naturally from the transcripts and ensuring they faithfully reflect the perspectives and experiences of the participants [35].

### 2.4. Interview Guide

The interview guide for this study was meticulously developed by A.D.F, A.R.O.M., and R.L.V. (Supplementary File S1); drawing upon insights from a prior quantitative survey [26] and a thorough review of the scientific literature. Unlike focusing solely on chronic pain management, the literature review encompassed a broader scope, delving into the challenges, obstacles, differences, and various aspects physiotherapists face in chronic pain management. The structure of the guide included both open-ended questions and targeted probing queries designed to elicit in-depth responses.

The initial interviews functioned as pilot tests, enabling the research team to refine the guide to ensure it thoroughly addressed the study’s objectives. Minor adjustments, such as rephrasing and reordering questions, were made to improve clarity and ensure the interview guide effectively captured the intended data. After reviewing their relevance and alignment with the research question, these pilot interviews were included in the final analysis. Therefore, the guide was utilized not just as a static tool but as a dynamic framework, allowing for adjustments and evolution based on the flow of each interview. This flexible approach facilitated the incorporation of emerging themes and insights from early interviews into subsequent ones, ensuring a rich and comprehensive exploration of the physiotherapists’ experiences, challenges, and perspectives in managing chronic pain.

### 2.5. Data Collection

The primary investigators—A.D.F., A.R.M.O., and R.L.V. —determined that 12 interviews would suffice to achieve the study’s objectives, adhering to the principles of information power [36]. The study aimed to explore the experiences, challenges, and facilitators encountered by physiotherapists in the management of chronic pain patients. The dialogues in the interviews were characterized by their high quality and specificity, contributing significantly to the depth of the study.

Data saturation was considered reached after the 12th interview, as no new themes or codes were identified in subsequent interviews, and responses became repetitive [37].

This aligns with established guidelines in qualitative research, which suggest that saturation typically occurs between the 10th and 15th interviews, particularly in studies with relatively homogeneous samples [38]. While additional interviews could potentially provide further confirmation, the redundancy in the data by the 12th interview indicated that we had sufficiently captured the range of perspectives necessary to address the research questions comprehensively. These interviews were conducted online via Google Meet, each lasting approximately 45 to 60 min, and carried out between February 2024 and March 2024. Participants had the opportunity to review their transcripts prior to the analysis, to ensure accuracy and validate their responses; following established practices in qualitative research [39].

### 2.6. Data Analysis

The transcriptions were uploaded into ATLAS.ti, 23.2.1 Mac version—a qualitative data analysis software—to facilitate systematic coding and organization of the data. Braun and Clarke’s [40,41] six-phase reflexive thematic analysis was followed. The coding process began with open coding, where initial codes were generated directly from the data, allowing for a broad exploration of themes. This method was followed by axial coding to identify relationships between the initial codes and develop broader themes, providing a more structured understanding of the data. Finally, selective coding was employed to refine and integrate the themes into coherent findings, ensuring that the core themes were well defined and thoroughly supported by the data. This combination of coding methods was chosen to systematically and comprehensively explore the complex experiences of physiotherapists managing chronic pain. Two researchers independently (E.O.G and I.C.P.) coded the data to enhance reliability, ensuring the coding process was consistent and unbiased. Discrepancies were discussed and resolved through regular consensus meetings, which provided a systematic approach to reconciling differences and ensuring a robust analysis. A third researcher (A.R.O.M), who is experienced in qualitative analysis, reviewed the final themes to ensure validity and coherence, enhancing the study’s rigor and helping to mitigate potential biases.

Triangulation techniques were employed to ensure the robustness of the findings further. Investigator triangulation was used, in which multiple researchers contributed to the analysis process. Specifically, two researchers independently coded the data, and discrepancies were discussed and resolved through consensus meetings. A third researcher reviewed the final themes to provide an additional layer of scrutiny and validation. This triangulation method helped to avoid the potential bias of the principal investigator, A.D.F., by incorporating multiple perspectives into the analysis.

The research team also engaged in reflexive practices, regularly reflecting on their potential biases and how they might influence the data interpretation. This process involved maintaining reflexive journals and regularly discussing the researchers’ roles and perspectives throughout the research process. By being aware of and addressing their biases, especially those of the principal investigator, the researchers aimed to ensure a more objective and balanced interpretation of the data.

In our study, triangulation techniques and reflexive practices work together to strengthen the credibility and reliability of the findings. While triangulation ensures that findings are validated through multiple sources and perspectives, reflexive practices ensure that the researchers’ potential biases and influences are acknowledged and managed throughout the research process.

Data saturation was determined to have been reached when no new themes or insights were emerging from the interviews. This point was reached after thorough and repeated data reviews, ensuring the analysis captured the full depth and breadth of the physiotherapists’ experiences.

## 3. Results

### 3.1. Participants

Characteristics of the 12 participating physiotherapists are presented in Table 1.

### 3.2. Broad Themes Synthesis

**Figure 1:**
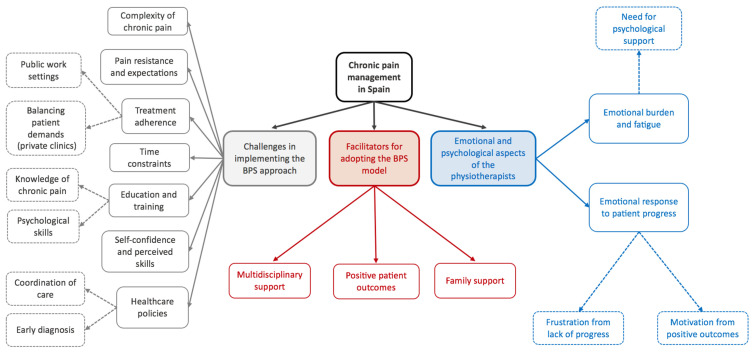
Thematic map of the results.

Three broad themes were generated from the data: (1) challenges in implementing the BPS approach, (2) facilitators for adopting the BPS model, and (3) emotional and psychological aspects of the physiotherapists. Figure 1 illustrates the thematic map of the results.

#### 3.2.1. Theme 1: Challenges in Implementing the Biopsychosocial Approach

Physiotherapists reported various challenges in implementing the BPS approach in their practice. These challenges stem from patient resistance, inadequate training, limited resources, and several other factors.

- Sub-theme 1.1: Complexity of chronic pain. The multifactorial nature of chronic pain makes it difficult to diagnose and treat effectively. Three physiotherapists noted that the complexity of chronic pain often requires a comprehensive approach that addresses multiple dimensions of the patient’s experience. One of them stated: “*Chronic pain is never straightforward. I try to stay updated on chronic pain management, but sometimes, it feels incomprehensible even to me. Explaining this to patients is challenging.*”—Physiotherapist 1 (Hospital).
- Sub-theme 1.2: Education and training. A lack of specific training in chronic pain management and advanced techniques was identified as a barrier. All of the physiotherapists expressed the need for more targeted education to implement the BPS approach effectively. Many reported feeling inadequately prepared to handle psychological aspects, such as motivating patients, dealing with flare-ups, and improving adherence through effective communication. Some representative quotes of these ideas are: “*I need more specialized training in chronic pain management to feel confident in using the BPS model. I admit it.*”—Physiotherapist 2 (Primary care). “*I often struggle when motivating patients to stick with their exercise routines. Sometimes I don’t know what to say to keep them engaged, despite their pain*”—Physiotherapist 10 (Private clinic). “*When patients have flare-ups, it’s hard to know the best way to support them psychologically. I just don’t get enough training in that area. I know I feel this way, and I’m sorry…*”—Physiotherapist 11 (Primary care). “*My biggest challenge is finding the right words to keep patients engaged and committed to the treatment plans. It’s tough to keep their spirits up and ensure they understand the importance of continuing, even when progress seems slow, and the pain doesn’t disappear.*”—Physiotherapist 9 (Hospital).
- Sub-theme 1.3: Self-confidence and perceived skills. Despite their theoretical knowledge, five physiotherapists reported a lack of self-confidence and perceived skills related to the BPS approach in certain situations. This issue was highlighted as a significant barrier to providing adequate care. Some representative examples are: “*I try to stay updated on chronic pain management, but there are moments in clinical practice, especially during patient flare-ups, when I feel unsure about how to proceed. It makes me feel unsure.*”—Physiotherapist 10 (Private clinic). “*I believe I treat my chronic pain patients well, giving them the time they need, explaining their condition, emphasizing active movement and self-care. […]. Yet, sometimes, I feel like something is missing in my methodology, something that would help me connect better with them. I can’t quite pinpoint what it is.*”—Physiotherapist 11 (Primary care). “*Even though I understand the BPS approach, there are times when I struggle to find the right words or actions to keep patients engaged and motivated. It’s frustrating and makes me doubt my capabilities.*”—Physiotherapist 12 (Primary care).
- Sub-theme 1.4: Patient resistance and expectations. Patient resistance to the BPS model is a significant challenge. All of the participant physiotherapists state that many patients expect fast solutions and have difficulty accepting the need for a comprehensive approach that includes psychological and social components and a return to normal movement and activity.

“*Some patients simply want a quick fix. They don’t understand why we need to address the psychological aspects of their pain. They need to understand that pain can persist and that they need to start moving and doing things they have stopped doing for so long.*”

—Physiotherapist 4 (Primary care).

“*Patients often come in asking for specific manual treatments, and shifting their mindset towards a more holistic approach is tough. They often don’t understand why we haven’t touched or treated them, especially in the first sessions when we try to make neuroscience education.*”

—Physiotherapist 7 (Private clinic).

- Sub-theme 1.5: Treatment adherence. Maintaining long-term treatment adherence is challenging for many patients. Factors such as the complexity of chronic pain, delayed treatment outcomes, and patient resistance all contribute to poor adherence to prescribed treatment strategies. Additionally, greater effort from physiotherapists is often required to engage and motivate these patients than those with other conditions.
  ○ Tactics for adherence in public work settings: Physiotherapists in public settings generally reported focusing more on adherence to home exercise programs and lifestyle changes rather than the immediate satisfaction of patients, as treatments are publicly funded and free of charge. “*In public practice, our main goal is to ensure patients follow their home exercise programs and lifestyle recommendations rather than worrying about them returning for paid sessions.*”—Physiotherapist 9 (Hospital).
  ○ Balancing patient demands in private clinics: Physiotherapists in private settings revealed unique pressures from patient expectations due to the direct payment model. They sometimes had to balance the BPS approach with meeting patient expectations to ensure satisfaction and return visits. “*Running a private clinic means I have to balance the BPS approach with what patients expect. They often want specific manual therapies because they’ve already tried many other treatments elsewhere […]. While I know the BPS model is effective, I also need to make sure they’re happy with the treatment so they keep coming back, even if it means occasionally providing treatments I don’t fully endorse. It’s difficult to maintain that equilibrium.*”—Physiotherapist 6 (Private clinic). “*In private practice, if a patient wants a specific treatment, it’s hard to convince them otherwise because they’re paying for it.*”—Physiotherapist 3 (Private clinic).
- Sub-theme 1.6: Time constraints. A lack of time was frequently mentioned as a barrier to implementing the BPS approach. High patient loads and administrative tasks further limit the time available for each patient. All physiotherapists acknowledged that time is a critical resource for these patients due to their high workload. Some examples are: “*The heavy administrative burden leaves little time to engage with the BPS approach fully.*”—Physiotherapist 5 (Hospital). “*We’re always so rushed […]. It’s hard to find enough time to spend with each patient, which is what they need most.*”—Physiotherapist 8 (Primary care).
- Sub-theme 1.7: Healthcare policies and practices:
  ○ Subtheme 1.7.1: Coordination of Care. Challenges in communication and coordination with other healthcare professionals involved in treating chronic pain patients were also noted as barriers. Effective interdisciplinary collaboration is essential but often lacking. “*The coordination, the real coordination with other professionals, is complicated. Sometimes we’re not all on the same page, which affects the patient’s treatment.*”—Physiotherapist 3 (Private clinic) “*I work in a hospital where a multidisciplinary team treats chronic pain patients, but we’re not coordinated. Appointments are on different days, and sometimes, our messages are contradictory […]. It’s like each of us has different objectives, and patients notice this and get confused. We need to change that; it’s so important.*”—Physiotherapist 12 (Hospital).
  ○ Subtheme 1.7.2: Importance of early diagnosis. Early diagnosis is critical in the management of chronic pain. Physiotherapists noted that many patients are referred to them after prolonged periods of ineffective treatments, often after multiple surgeries or interventions. This delay exacerbates the patient’s condition and complicates the treatment process due to increased psychological and emotional burdens. Early diagnosis and intervention can prevent the escalation of chronic pain, leading to more effective management and better patient outcomes. This appreciation highlights the need for improved screening processes and early referral practices within the healthcare system, to ensure timely and appropriate care for chronic pain patients.

Several relevant quotes illustrate these points:

“*It’s so frustrating when patients come to us as a last resort. […]. They’ve been through so much already: multiple surgeries or endless medications. Early intervention would make such a difference, but by the time we see them, the pain has taken a huge toll on their mental health too.”- (Physiotherapist 5, Hospital). “We often get patients who have bounced around different specialists and treatments for years. If we could catch them earlier, we could help manage their pain more effectively and prevent it from becoming this overwhelming, chronic issue. Early diagnosis is key, but it’s not happening enough.*”

—(Physiotherapist 3, Private clinic).

#### 3.2.2. Theme 2: Facilitators for Adopting the BPS Model

Despite the challenges, specific facilitators can enhance the adoption of the BPS model, such as multidisciplinary support and observing positive patient outcomes.

- Sub-theme 2.1: Multidisciplinary support. Effective multidisciplinary collaboration is crucial for implementing the BPS approach. Most physiotherapists highlighted the importance of working with other healthcare professionals to provide comprehensive care. Two physiotherapists working in a hospital setting are examples of this aspect: “*Working closely with psychologists and occupational therapists has significantly improved our patients’ outcomes. Coordinated efforts ensure that we address all aspects of their pain, which leads to more effective treatment for them.*”—Physiotherapist 1 (Hospital). “*In our hospital, the multidisciplinary team is well-coordinated, and it makes a huge difference. Patients receive consistent messages and a comprehensive treatment plan that addresses their physical, psychological, and social needs.*”—Physiotherapist 12 (Hospital).
- Sub-theme 2.2: Positive patient outcomes. Seeing positive outcomes in patients who adhere to the BPS model strongly motivates physiotherapists. Successful cases reinforce the value of the BPS approach and encourage its continued use, despite the initial challenges. Three physiotherapists noted this issue. One said: “*When patients understand and engage with the BPS approach, their progress is remarkable. It’s worth the effort. Seeing them recover their independence and improve their quality of life is incredibly rewarding.*”—Physiotherapist 8 (Primary care). Another physiotherapist said: “*I’ve seen significant improvements in patients who follow the BPS model, which is very encouraging. It encourages me that we’re on the right track and motivates me to continue using this approach.*”—Physiotherapist 7 (Private clinic).
- Sub-theme 2.3: Family support. Family support is crucial in enhancing treatment adherence by providing additional motivation for patients. Family members often play a critical role in encouraging and supporting patients to follow their treatment plans, making the family’s involvement essential for long-term success. “*Having the family involved is key. Their support can really push the patient to stay on track with their exercises and lifestyle changes. It’s a big part of why some patients succeed in the long run.*”—Physiotherapist 5 (Hospital).

Several physiotherapists also noted that addressing the existing challenges would inherently facilitate adoption of the BPS model. Implementing the BPS approach could become more effective and widespread by improving areas such as training, resource availability, and interdisciplinary coordination.

#### 3.2.3. Theme 3: Emotional and Psychological Aspects 

A significant portion of the physiotherapists (four of twelve) interviewed reported experiencing emotional and psychological challenges in their practice with chronic pain patients. These challenges often arise from the complexity and chronic nature of the pain conditions they manage. There is notable variability in how physiotherapists experience these challenges, with some finding motivation in positive patient outcomes. In contrast, others feel frustrated when progress is limited, indicating that not all practitioners face the same emotional burden.

- Subtheme 3.1: Emotional burden and fatigue: Two physiotherapists expressed that managing chronic pain patients leads to significant emotional burden and exhaustion. This emotional strain frequently stems from the constant need to provide both physical and emotional support to patients with chronic pain. As one physiotherapist mentioned, “Managing chronic pain patients can be mentally exhausting; it often feels like it drains your energy, you know?”—Physiotherapist 3 (Private clinic).
- Subtheme 3.2: Need for psychological support: The need for psychological support and coping strategies for physiotherapists was a repeated theme; two highlighted the overwhelming emotional strain and the necessity for better psychological support systems for healthcare providers. One physiotherapist emphasized, “Sometimes, the emotional strain of dealing with chronic pain patients is overwhelming to me. Honestly, I think we need better psychological support systems for healthcare providers” —Physiotherapist 6 (Private clinic).
- Subtheme 3.3: Emotional response to patient progress

3.3.1: Motivation from positive outcomes. Despite the emotional challenges, two physiotherapists found motivation in the positive outcomes of their patients. The gratitude expressed by patients and their improvements in condition were significant sources of motivation for the physiotherapists. One physiotherapist noted, “*It’s really rewarding when a patient tells you they feel better, can move more, or thanks you for helping them become more independent. It makes it all worth it. […]. It’s always gratifying when a patient improves, you know, but it’s especially satisfying and comforting with these complex chronic pain patients where you often don’t even know where to start*”

—Physiotherapist 8 (Private clinic).

3.3.2: Frustration from lack of progress. Conversely, two physiotherapists experienced frustration when patients did not show improvement, despite their efforts. This frustration was particularly pronounced among those who had confidence in their training and dedication to managing chronic pain but faced setbacks when expected outcomes were not achieved. One of them stated: “*Even though I feel well-prepared and passionate about treating chronic pain, it’s frustrating when patients don’t show progress. It can sometimes be disheartening*”

—Physiotherapist 4 (Primary care).

In addition to the individual themes and sub-themes identified, several interrelationships emerged from the data analysis, highlighting the complex interplay between various factors influencing physiotherapists’ experiences and practices in managing chronic pain. These relationships were identified through a comprehensive analysis of the interview transcripts; reflecting the interconnected nature of the challenges, facilitators, and emotional aspects described by the Spanish physiotherapists. This analysis revealed several key relationships:

○ Positive patient outcomes and motivation from positive outcomes: Positive patient outcomes are a significant motivator for physiotherapists, reinforcing their commitment to the BPS model. When patients show improvement, physiotherapists feel validated in their approach and are encouraged to continue using the BPS model, despite the challenges.
○ Self-confidence and perceived skills: The lack of self-confidence and perceived skills are closely linked to the emotional and psychological aspects of physiotherapists, as well as the complexity of chronic pain and limited training. Physiotherapists who feel unsure of their skills are more likely to experience emotional strain and frustration, particularly when dealing with complex chronic pain cases.
○ Coordination of care and multidisciplinary support: Effective care coordination is crucial in chronic pain management. Poor coordination can undermine these efforts, making it harder to provide comprehensive care to patients with chronic pain.
○ Patient resistance and treatment adherence: Patient resistance and expectations directly impact treatment adherence, necessitating strategies to manage these challenges. Physiotherapists must often balance addressing patient expectations with educating them about the BPS model to improve adherence.
○ Complexity of chronic pain and time constraints: The multifactorial nature of chronic pain often requires more time for proper management, exacerbating time constraints. This difficulty creates additional pressure on physiotherapists to manage their time effectively, while providing comprehensive care.
○ Family support and treatment adherence: Strong family support enhances treatment adherence by providing additional motivation for patients. Family members often play a crucial role in encouraging and supporting patients to adhere to their treatment plans, highlighting the importance of involving families in the care process.

For more detailed information and representative quotes from these relationships found among the physiotherapist’ narratives, see Supplementary Table S1.

## 4. Discussion

This study aimed to investigate the experiences and challenges physiotherapists face in Spain when managing chronic pain across different healthcare settings. It provides a novel contribution to understanding the lived experiences of physiotherapists dealing with chronic pain, while also exploring the BPS approach in the context of Spanish physiotherapy practice. Unlike previous studies, which have predominantly focused on broader international contexts, this research explores explicitly the unique challenges and facilitators faced by physiotherapists in Spain. Using a qualitative approach, we delved into the intricacies of their daily practices and uncovered significant insights. The main findings of this research highlighted three broad themes: (1) challenges in implementing the biopsychosocial (BPS) approach, (2) facilitators for adopting the BPS model, and (3) emotional and psychological aspects of the physiotherapists. These themes offer a comprehensive understanding of the multifaceted issues physiotherapists encounter. Some challenges include patient resistance, inadequate training, and time constraints, which hinder the effective implementation of the BPS model. On the other hand, key facilitators such as multidisciplinary support and observing positive patient outcomes were significant. Notably, the study also revealed the distinct pressures physiotherapists face in private clinics compared to their counterparts in public settings. Lastly, the emotional and psychological toll on physiotherapists underscores the need for better support systems to help them manage these stresses.

The findings are particularly significant as they provide a detailed view of the cultural and systemic factors influencing chronic pain management in Spain. This perspective has been underrepresented in the literature. This study’s focus on the emotional and psychological burdens on physiotherapists also adds a new dimension to understanding the professional challenges in this field, highlighting the necessity for targeted support mechanisms.

### 4.1. Challenges in Implementing the Biopsychosocial Approach

Chronic pain presents significant challenges due to its complex interaction of biological, psychological, and social factors; as highlighted by the physiotherapists in this study. This complexity requires a comprehensive biopsychosocial approach, as supported by recent literature, which advocates for collaborative models of care to improve patient outcomes and care delivery [42,43].

Addressing this complexity requires physiotherapists to have adequate training in both chronic pain management and psychological techniques. Studies have shown that physiotherapists often feel inadequately prepared to handle the psychological aspects of chronic pain management, such as patient motivation, dealing with flare-ups, or effective communication [16,17,18,24,43]. This gap in training hinders their ability to implement the biopsychosocial model fully, highlighting the need for enhanced educational programs that equip physiotherapists with the necessary skills and up-to-date knowledge about chronic pain [16,17,18].

This lack of sufficient training directly impacts physiotherapists’ self-confidence and perceived skills, creating another significant barrier to the effective implementation of the BPS approach. Previous studies in Spain have highlighted that physiotherapists often report moderate confidence and skill levels, which are insufficient to comprehensively apply the BPS model [42]. In another study conducted in Spain, almost 65% of physiotherapists identified inadequate psychological skills as a primary barrier, and nearly 10% consistently applied the BPS approach in their practice [26]. These findings underscore the critical need for targeted training programs to bridge this gap and comprehensively enhance physiotherapists’ self-confidence and perceived skills in managing chronic pain. This need for enhanced training is also supported by broader literature, which consistently identifies insufficient training and resources as significant barriers to the effective adoption of the BPS model [16,17,18].

Patient resistance to and expectations of the BPS model, and challenges in maintaining treatment adherence are significant barriers in chronic pain management. Many patients expect quick fixes and have difficulty accepting the need for a comprehensive approach that includes psychological and social components [16,44]. This resistance is often due to deeply ingrained beliefs about pain and treatment expectations, shaped by previous healthcare experiences. Studies have shown that patient education and effective communication are critical in addressing these misconceptions and fostering a more holistic understanding of pain management [7,18,23,43].

Maintaining long-term treatment adherence is further complicated by these patient expectations. The complexity of chronic pain, delayed treatment outcomes, and patient resistance contributes to poor adherence to prescribed treatment strategies. Physiotherapists often need to exert additional effort to engage and motivate patients, emphasizing the importance of patient education and the gradual integration of BPS principles and movement [16,17,18,43]. In public settings, physiotherapists focus on adherence to home exercise programs and lifestyle changes, without the immediate pressure of patient satisfaction for return visits [24]. In contrast, private practice physiotherapists must balance the BPS approach with meeting patient demands, to ensure satisfaction and return visits, which can lead to compromised adherence tactics [23,24]. This dynamic highlights the necessity for strategies that foster patient understanding and adherence to the BPS model across different healthcare settings, ultimately improving chronic pain management outcomes [16,17].

Time constraints are an omnipresent barrier to effectively implementing the BPS model in chronic pain management. High patient loads and administrative tasks significantly limit the time available for each patient, making it challenging for physiotherapists to fully engage with the BPS approach [16,17,18,19]. The heavy administrative burden often leaves little time for thorough patient interaction and comprehensive care. This challenge is exacerbated by the complexity of chronic pain, which requires significant time for proper assessment and intervention [16,17]. To mitigate this issue, healthcare systems must address workload management and streamline administrative processes, allowing physiotherapists to dedicate more time to patient care [43].

Furthermore, healthcare policies and practices significantly impact the implementation of the BPS approach. Effective care coordination is crucial for managing chronic pain, yet many physiotherapists report communication and interdisciplinary collaboration challenges. These coordination issues can result in inconsistent messages to patients and fragmented care [16,17]. Improved multidisciplinary collaboration is essential for comprehensive care and better patient outcomes [43].

Early diagnosis and intervention are also critical components of effective chronic pain management. Delays in referral and treatment often exacerbate conditions and increase patient psychological and emotional burdens. This was also emphasized by several physiotherapists in the interviews, who noted that delayed referrals often complicate treatment outcomes and increase patient distress. This urgency highlights the need for improved screening processes and early referral practices within the healthcare system, to ensure timely and appropriate care for chronic pain patients [44]. Early intervention can prevent the escalation of chronic pain, leading to more effective management and better patient outcomes [45,46]. Triage processes at multidisciplinary pain clinics have been highlighted as critical in reducing wait times and ensuring optimal access to care, though many clinics face challenges with their current systems [47]. These findings collectively reinforce the necessity of structured and effective diagnostic triage and early intervention strategies in chronic pain management [16,17,18].

By addressing these barriers and implementing targeted strategies—such as enhanced training, patient education, and improved healthcare policies—adopting the BPS model in chronic pain management can be significantly improved, leading to better outcomes for both patients and physiotherapists.

### 4.2. Facilitators for Adopting the BPS Model

Despite these challenges, the study identified key facilitators that can enhance the adoption of the BPS model. Effective multidisciplinary collaboration emerged as a crucial element, aligning with numerous reviews’ findings [16,17,18]. Physiotherapists in our study highlighted the importance of working closely with other healthcare professionals—such as psychologists, general practitioners, or occupational therapists—to provide holistic care. This effective collaboration involves setting shared goals and respecting each professional’s unique role without overstepping boundaries. This collaborative approach ensures that all aspects of the patient’s pain experience are addressed, leading to better patient outcomes and reinforcing the value of the BPS model.

Positive patient outcomes also serve as a significant motivator for physiotherapists. Seeing tangible improvements in their patients’ conditions reinforces the effectiveness of the BPS approach and encourages its continued use. This finding is consistent with previous research that emphasizes the impact of patient progress on clinician motivation [16].

Family support is crucial in influencing treatment adherence and patient outcomes in chronic pain management. Physiotherapists in this study noted that family involvement can significantly boost patients’ motivation and adherence to treatment plans, aligning with previous literature on the positive impact of social support [17,23,43]. However, the literature also indicates that unsupportive families, who are either frustrated or overly protective, may inadvertently reinforce maladaptive pain behaviours or encourage dependency, complicating treatment [7]. Research has demonstrated that patients from supportive families report less pain intensity, reduced reliance on medication, and higher activity levels [48]. Thus, physiotherapists should engage families in the treatment process, educating them on promoting active coping strategies and patient independence [49].

It should be noted that several physiotherapists mentioned that addressing the existing challenges could inherently act as facilitators for adopting the BPS model. Improving areas such as training, resource availability, and interdisciplinary coordination could make implementing the BPS approach more effective and widespread.

### 4.3. Emotional and Psychological Aspects of the Physiotherapists

The emotional and psychological burden on physiotherapists is a significant finding of this study. Managing chronic pain patients often leads to emotional exhaustion and the need for psychological support. This result aligns with Ng et al. (2022) and Holopainen et al. (2018), who also identified emotional strain as a critical issue for healthcare providers. Additionally, the variability in emotional responses—ranging from motivation when witnessing positive outcomes, to frustration when progress is limited—highlights the complex emotional landscape in chronic pain management [50]. The novelty of our study lies in revealing the demand for training in the psychological aspects of patient care and self-care strategies for physiotherapists. This finding underscores the necessity of institutional support mechanisms, such as counselling services and peer support groups, to mitigate the psychological burden on physiotherapists [51].

### 4.4. Interrelationships among Themes

The study also revealed several interrelationships among the identified themes, reflecting the interconnected nature of the challenges, facilitators, and emotional aspects of managing chronic pain. For instance, positive patient outcomes significantly enhance physiotherapists’ motivation and commitment to the BPS model [16]. Similarly, the lack of self-confidence and perceived skills is closely linked to the emotional strain experienced by physiotherapists [18]. Effective care coordination and multidisciplinary support are crucial for addressing patient resistance and improving treatment adherence [43]. Recognizing these interrelationships is essential for developing comprehensive strategies to enhance chronic pain management [17].

This study has certain limitations, including a relatively small sample size, and its regional focus on Andalucía, which may limit the generalizability of the findings. The reliance on self-reported data may also introduce bias, as participants might underreport or overestimate their experiences and challenges. Additionally, the qualitative nature of the study, while providing in-depth insights, does not allow for statistical generalization to broader populations. These limitations should be considered when interpreting the results and their applicability to broader contexts. Future research with larger, more diverse samples and incorporating quantitative methods could help validate and extend these findings.

The findings of this study have several significant implications for clinical practice. First, there is a pressing need for enhanced training programs that equip physiotherapists with the comprehensive skills required to implement the BPS model effectively. This includes training in the psychological aspects of chronic pain management and developing solid interdisciplinary collaboration skills. Second, healthcare systems, especially those in private settings, must recognize the unique pressures physiotherapists face and provide adequate support to balance patient expectations with best clinical practices. Third, the importance of early diagnosis and intervention cannot be overstated, as timely management can prevent the escalation of chronic pain and improve patient outcomes. Additionally, leveraging family support effectively is crucial for enhancing treatment adherence and patient outcomes. Family members should be educated on promoting active coping strategies and supporting patient independence. Finally, the considerable emotional and psychological burden on physiotherapists underscores the necessity for robust support systems, such as counselling services and peer support groups, to help them manage these stresses effectively, as well as provide them with psychological support and tools to combat demotivation and maintain adherence to best practices in patient care.

## 5. Conclusions

This study provides valuable insights into the challenges and facilitators of adopting the BPS model among physiotherapists in Spain. Key barriers identified include patient resistance to the BPS model, inadequate psychological training, difficulties in interdisciplinary collaboration, time constraints, and the complexity of chronic pain management. Additionally, the emotional and psychological burden on physiotherapists working with chronic pain patients was highlighted as a significant challenge.

Facilitators that support the adoption of the BPS model include effective multidisciplinary collaboration, positive patient outcomes, and family involvement; all of which were reported to enhance patient adherence and overall treatment success. Addressing these challenges through improved psychological training, institutional support, and better coordination among healthcare providers—alongside fostering early diagnosis and stronger family involvement—can enhance the adoption of the BPS model. This comprehensive approach can ultimately improve outcomes for patients with chronic pain and increase professional satisfaction among physiotherapists.

Addressing these challenges through targeted psychological training, institutional support, and improved interdisciplinary collaboration, along with involving families more closely in patient care, can enhance the adoption of the BPS model. This comprehensive approach can ultimately improve outcomes for patients with chronic pain and increase professional satisfaction among physiotherapists.

## Figures and Tables

**Table 1:** Sociodemographic characteristics of participating physiotherapists.

Participant	Age (Years)	Gender	Education Level	Clinical Experience (Years)	Work Setting	Trained in CP Management	Type of CP Patients Most Treated
1	35	Female	Master	14	Hospital	Yes	Mixed
2	32	Male	Master	11	Primary care	Yes	Chronic back pain
3	49	Female	PhD	28	Private clinic	Yes	Mixed
4	50	Male	Master	25	Primary care	Yes	Musculoskeletal CP
5	44	Female	Master	21	Hospital	Yes	Mixed
6	39	Male	PhD	17	Private clinic	Yes	Mixed
7	54	Female	PhD	33	Private clinic	Yes	Musculoskeletal CP
8	37	Male	Master	16	Primary care	Yes	Mixed
9	45	Female	Master	22	Hospital	Yes	Chronic back pain
10	34	Male	Master	12	Private clinic	Yes	Musculoskeletal CP
11	46	Female	Master	25	Primary care	Yes	Fibromyalgia
12	53	Female	Master	31	Hospital	Yes	Mixed

Abbreviations: CP, chronic pain; PhD, Doctor of Philosophy in Physical Therapy.

## Data Availability

Data used to support the findings of this study are available from the corresponding author upon request.

## Supplementary Materials

The following supporting information can be downloaded at: https://www.mdpi.com/article/10.3390/clinpract14050165/s1, Supplementary Table S1. Interrelation between key themes and subthemes. Supplementary File S1. Interview guide.
