# Supplementary material for: Chronic Pain in Spanish Physiotherapy Practice: Treatment Challenges and Opportunities in Diverse Healthcare Settings—A Qualitative Study
Source: Clin Pract. 2024 Oct 10;14(5):2089–104. doi: 10.3390/clinpract14050165 (PMC11505835; doi:10.3390/clinpract14050165)
Supplement: Supplementary file 1 [file clinpract-14-00165-s001.zip › Supplementary File S1. Semi-structured interview.pdf]

SUPPLEMENTARY FILE S1: GUIÓN ENTREVISTA SEMI-ESTRUCTURADA con FISIOTERAPEUTAS ESPECIALISTAS EN TRATAMIENTO DE PACIENTES CON DOLOR CRÓNICO

NÚMERO ENTREVISTA:

Entrevistador/a:

Fecha:

Hora inicio:

Hora fin:

Buenos días/tardes: Mi nombre es Ángeles Díaz Fernández, investigadora en la Universidad de Jaén. En el marco de un estudio cualitativo sobre las experiencias de fisioterapeutas en el tratamiento de pacientes con dolor crónico, le agradezco sinceramente su disposición a participar en esta entrevista.

Hoy nos centraremos en explorar y comprender en profundidad los retos, obstáculos y factores facilitadores que usted, como profesional de la fisioterapia, encuentra al tratar pacientes con esta condición. Su perspectiva es esencial para enriquecer nuestro conocimiento en este campo y para identificar posibles áreas de mejora en la práctica clínica.

Durante nuestra conversación, abordaremos temas relacionados con su experiencia profesional, incluyendo las estrategias de tratamiento, los desafíos específicos que enfrenta, y cómo estos impactan en su práctica diaria y en su percepción sobre la eficacia del tratamiento.

Es importante para nosotros conocer tanto sus experiencias personales como sus opiniones profesionales. No hay respuestas correctas o incorrectas; valoramos profundamente su honestidad y su punto de vista único.

Le recuerdo que esta sesión será grabada para asegurar que no se pierda ningún detalle importante de lo que comparta. Sin embargo, la confidencialidad es una prioridad para nosotros: sus respuestas serán anonimizadas y no se le identificará personalmente en los resultados finales del estudio.

Deseo que se sienta libre de expresar su opinión con franqueza y confianza. Aunque tengo varias preguntas preparadas, es posible que en algunos momentos le pida que sea breve o que interrumpa para pasar a la siguiente pregunta, con el fin de abarcar todos los temas relevantes en el tiempo disponible.

Antes de comenzar, le pediré que lea y firme el consentimiento informado, que explica en detalle el propósito y la naturaleza de este estudio. Una vez hecho esto, procederemos con una breve presentación suya, seguida de las preguntas de la entrevista.

Nuevamente, gracias por su tiempo y su valiosa contribución a esta investigación. Su experiencia y conocimientos son fundamentales para avanzar en el entendimiento y la mejora del tratamiento del dolor crónico en el campo de la fisioterapia.

Experiencia / contexto laboral y conocimientos del fisioterapeuta

1. ¿Has tratado a menudo a pacientes con dolor crónico? ¿Crees que son pacientes más complejos o no, igual que el resto de pacientes?
2. ¿Trabajas en el sector público o privado? ¿Crees que hay diferencias en el tratamiento a este tipo de pacientes dependiendo de dónde se trabaje?
3. ¿Crees que tienes un conocimiento adecuado sobre la moderna neurociencia del dolor?
4. ¿Qué papel crees que tienen los fisioterapeutas en el tratamiento del dolor crónico?

5. ¿Conoces la actual recomendación de tratamiento con orientación biopsicosocial a los pacientes con dolor crónico?
6. ¿Te suenan conceptos como PSE/PNE, Reestructuración cognitiva, Terapia Cognitivo Conductual, Mindfulness, Terapia de Aceptación y Compromiso o Flexibilidad Psicológica? ¿Sabes utilizarlos como técnicas de tratamiento? ¿Los has utilizado con tus pacientes que sufren de dolor crónico?
7. ¿Cómo te mantienes actualizado en las últimas investigaciones y tratamientos para el dolor crónico?

Percepciones y actitudes hacia el tratamiento

1. ¿Crees que ofreces un adecuado tratamiento a los pacientes con dolor crónico? ¿Te sientes capacitado para tratar a los pacientes con dolor crónico? ¿Encuentras limitaciones a la hora de tratar a este tipo de pacientes? ¿Cuáles son?
2. ¿Qué confianza tienes en ti y en tus capacidades al tratar a pacientes en el que siempre existe dolor?
3. ¿Cuando llegan a ti están en las primeras fases/llevan poco tiempo o al contrario, ya llevan mucho tiempo sufriendo de dolor crónico? ¿Qué te parece el tema del diagnóstico precoz del dolor crónico? ¿Qué opinas sobre un posible triaje a este tipo de pacientes?
4. ¿Qué te parece el tema de los recursos (tanto de tiempo o materiales) a la hora de tratar a estos pacientes?

Relación con los pacientes y con otros profesionales

1. ¿Qué piensas de la diferenciación de roles de los distintos profesionales, sobre todo me refiero al fisioterapeuta y al psicólogo? ¿Intrusismo?
2. ¿Cómo es tu relación con otros profesionales que también tratan a tus pacientes de dolor crónico, cómo es ese entorno laboral?
3. ¿Qué te parecen las expectativas de los pacientes con dolor crónico cuando acuden a la consulta del fisioterapeuta? ¿A veces estas expectativas del paciente han marcado el tratamiento que les has realizado/han dirigido ellos el tratamiento?
4. ¿Entienden los pacientes lo que realmente les pasa? ¿Cómo se lo explicas? ¿Qué haces con los pacientes que a pesar de explicárselo no lo entienden o no lo quieren entender, cómo enfocas el tratamiento entonces?
5. ¿Y sobre la alianza / relación terapéutica con estos pacientes? ¿Qué pasa cuando ellos esperan la disminución del dolor pero ésta no ocurre, qué ocurre con la confianza que depositan en nosotros?

Tratamiento y estrategias

1. ¿Qué puedes decirme del tratamiento multidisciplinar con este tipo de pacientes? ¿Trabajas en un equipo multidisciplinar?
2. Según tu punto de vista, ¿qué profesionales serían imprescindibles en un equipo multidisciplinar que trabajara con pacientes de dolor crónico?
3. ¿Qué puedes decirme de la medicación o sobremedicación de este tipo de pacientes?
4. ¿Y la familia, te parece que es facilitadora o una barrera en el tratamiento de estos pacientes? ¿Y el contexto social del paciente?
5. ¿Qué te parece la disyuntiva "Hands on/ Hands off"; cuál es tu perspectiva al respecto?
6. ¿Cuáles son tus estrategias principales a la hora de tratar a pacientes con dolor crónico?
7. ¿Cuáles crees que son tus puntos fuertes a la hora de tratar a pacientes con dolor crónico? ¿Y tus puntos débiles?
8. ¿Cómo manejas las expectativas de los pacientes respecto a la curación o mejora del dolor crónico?
9. ¿Cómo influye tu experiencia personal en el abordaje del dolor crónico?

Reflexiones personales y posibles mejoras

1. ¿Te consideras satisfecho en general con el tratamiento que haces del dolor crónico a tus pacientes? ¿Por qué?

2. ¿Cuáles son tus emociones al tratar a este tipo de pacientes? Sientes a veces frustración / decepción al tratar a estos pacientes o al contrario, te sientes motivado? ¿En qué sentido? ¿Por qué?
3. ¿Qué echas en falta a la hora de poder tratar a este tipo de pacientes tanto a nivel: personal, por parte de los pacientes o por parte de las instituciones?
4. ¿Cómo influye la política y regulación sanitaria actual con este tipo de pacientes?
5. ¿Crees que hacemos algo mal en el tratamiento de este tipo de pacientes? ¿Qué quitarías/pondrías / mejorarías?
6. ¿Algo más que quieras decir con respecto al tema?

Con esto damos por finalizada la sesión. Muchas gracias por haber participado en esta entrevista, tu colaboración es primordial para generar un acercamiento y análisis de resultados que pueda colaborar en las mejora del tratamiento a los pacientes con dolor crónico por parte de los fisioterapeutas.
