# Supplementary material for: Chronic Pain in Spanish Physiotherapy Practice: Treatment Challenges and Opportunities in Diverse Healthcare Settings—A Qualitative Study
Source: Clin Pract. 2024 Oct 10;14(5):2089–104. doi: 10.3390/clinpract14050165 (PMC11505835; doi:10.3390/clinpract14050165)
Supplement: Supplementary file 1 [file clinpract-14-00165-s001.zip › Supplementary Table S1. Interrelations themes_quotes.pdf]

Supplementary Table S1. Interrelationships between key themes and subthemes

**Interrelationship**	**Description**	**Example quotes**
Positive patient outcomes and motivation from positive outcomes	Positive patient outcomes boost physiotherapists' commitment to the BPS model. Improvement validates their approach and encourages continued use.	☙ *"When I see a patient finally making progress, it just fuels me to keep going with the BPS model, you know? It's like, all the effort is worth it."* (Physiotherapist 8, Primary care).<br>☙ *"It's so rewarding when patients tell me they feel better. It reassures me that the BPS approach is the right path. Keeps me motivated, for sure."* (Physiotherapist 7, Private clinic).
Self-confidence and perceived skills	Lack of self-confidence and skills is linked to emotional strain, chronic pain complexity, and limited training, leading to frustration.	☙ *"I stay updated on chronic pain management, but sometimes, during flare-ups, I feel lost about what to do next. It shakes my confidence a bit."* (Physiotherapist 10, Private clinic).<br>☙ *"I give my best to explain everything to my patients, but there are moments when I feel like something's missing in my approach.[…] It's frustrating."* (Physiotherapist 11, Primary care).<br>☙ *"Even with all the training, there are times when I struggle to keep patients engaged and motivated. Makes me question my skills sometimes."* (Physiotherapist 12, Primary care).

Coordination of care and multidisciplinary support	Effective care coordination is crucial for multidisciplinary support. Poor coordination can undermine efforts, complicating comprehensive care.	↪ *"Coordinating with other professionals in a hospital is really challenging. However, when we achieve it, the improvement in patient care is significant. It's great to see the impact on both the patients and our work efficiency."* (Physiotherapist 1, Hospital).<br>↪ *"Initially, getting our multidisciplinary team to work together was tough. But once we managed to synchronize our efforts, the treatment outcomes for chronic pain patients improved drastically. […]It was beneficial for the patients and made our jobs easier too."* (Physiotherapist 12, Hospital).
Patient resistance and treatment adherence	Patient resistance impacts treatment adherence. Balancing patient expectations with education on the BPS model improves adherence.	↪ *"When patients finally start to understand their pain and let go of some of their misconceptions, it's like a light bulb goes off. They become much more engaged in their treatment, and we see better adherence to the exercises and lifestyle changes we recommend. This really strengthens the therapeutic alliance between us."* (Physiotherapist 4, Primary care).<br>↪ *"It's amazing how patient education can turn things around. Once we manage to change their beliefs about pain, they are more willing to follow through with the treatment plan. This makes a huge difference in their*

		progress and our ability to help them, solidifying our therapeutic alliance." (Physiotherapist 2, Primary care).
Complexity of chronic pain and time constraints	Chronic pain complexity demands more time, increasing time constraints and pressure on physiotherapists for comprehensive care.	◦ *"Chronic pain is so multifaceted. You need time to address all aspects, but with a heavy workload, it's nearly impossible."* (Physiotherapist 1, Hospital).<br>◦ *"We're always rushed. Finding enough time to spend with each patient, especially those with complex pain, is a real challenge."* (Physiotherapist 8, Primary care).
Family support and treatment adherence	Strong family support enhances adherence, motivating patients to stick to treatment plans. Involving families is crucial for success.	*"For me, it's crucial to know the family and see the support they can provide to the patient. It's essential not just for pain relief, but to encourage movement and lifestyle changes. The family's involvement makes a significant difference in the overall success of the treatment and helps ensure that the patient maintains these habits in the long term, even when the pain persists."* (Physiotherapist 2, Primary care).
